# Pancreatic Cancer Research beyond DNA Mutations

**DOI:** 10.3390/biom12101503

**Published:** 2022-10-17

**Authors:** Hiromichi Sato, Kazuki Sasaki, Tomoaki Hara, Yoshiko Tsuji, Yasuko Arao, Chihiro Otsuka, Yumiko Hamano, Mirei Ogita, Shogo Kobayashi, Eric di Luccio, Takaaki Hirotsu, Yuichiro Doki, Hidetoshi Eguchi, Taroh Satoh, Shizuka Uchida, Hideshi Ishii

**Affiliations:** 1 Department of Medical Data Science, Center of Medical Innovation and Translational Research, Osaka University Graduate School of Medicine, Suita, Yamadaoka 2-2, Osaka 565-0871, Japan; 2 Department of Gastrointestinal Surgery, Osaka University Graduate School of Medicine, Suita, Yamadaoka 2-2, Osaka 565-0871, Japan; 3 Hirotsu Bio Science Inc., Chiyoda-Ku, Tokyo 102-0094, Japan; 4 Center for RNA Medicine, Department of Clinical Medicine, Aalborg University, Frederikskaj 10B, 2. (Building C), 2450 Copenhagen SV, Denmark

**Keywords:** pancreatic ductal adenocarcinoma, sequencing, mutations, RNA, cancer metabolism

## Abstract

Pancreatic ductal adenocarcinoma (PDAC) is caused by genetic mutations in four genes: KRAS proto-oncogene and GTPase (*KRAS*), tumor protein P53 (*TP53*), cyclin-dependent kinase inhibitor 2A (*CDKN2A*), and mothers against decapentaplegic homolog 4 (*SMAD4*), also called the big 4. The changes in tumors are very complex, making their characterization in the early stages challenging. Therefore, the development of innovative therapeutic approaches is desirable. The key to overcoming PDAC is diagnosing it in the early stages. Therefore, recent studies have investigated the multifaced characteristics of PDAC, which includes cancer cell metabolism, mesenchymal cells including cancer-associated fibroblasts and immune cells, and metagenomics, which extend to characterize various biomolecules including RNAs and volatile organic compounds. Various alterations in the *KRAS*-dependent as well as *KRAS*-independent pathways are involved in the refractoriness of PDAC. The optimal combination of these new technologies is expected to help treat intractable pancreatic cancer.

## 1. Introduction

Cancer is caused by numerous mutations in oncogenes and tumor suppressor genes [1,2]. Although pancreatic ductal adenocarcinoma (PDAC) has unique characteristics involving some driver mutations, a study on the multistep carcinogenesis of colorectal cancer revealed that cancer can be caused by the accumulation of “damages” in genes and by abnormalities in one or more driver genes, leading to an accumulating effect [3,4]. In multistep carcinogenesis, in the early stages of cancer, the accumulation of abnormalities, such as genomic deletions, in at least 7–8 genes because of long-term exposure to carcinogens is believed to trigger cancer occurrence; however, new mutations occur over the course of tumor development [3,4]. Current studies indicate that genes in all human genomes are significantly altered via mutations in cancer [3,4]. In contrast, initial mutations during PDAC development occur in a driver gene, KRAS proto-oncogene and GTPase, (*KRAS*) [5]. Another recent study indicated that the big 4 driver mutations commonly occur in PDAC (https://portal.gdc.cancer.gov (accessed on 1 August 2022)): (1) substitutions or alterations of nucleotides in *KRAS*; (2) tumor protein P53 (*TP53*); (3) cyclin-dependent kinase inhibitor 2A (*CDKN2A*); (4) mothers against decapentaplegic homolog 4 (*SMAD4*) (https://cancer.sanger.ac.uk/census (accessed on 1 August 2022)) [5]. Among these big 4 genes, mutations in at least *KRAS* and *TP53* help predict the survival of patients with resected PDAC [5]. A previous study investigated whether *KRAS* mutations influence persistent organic pollutants (POPs) and trace elements in PDAC survivors, which would indicate that KRAS mutation, POPs, and trace elements are not adversely related to PDAC survival when treatment was considered simultaneously [6]. This previous study determined that higher concentrations of metals, such as lead, cadmium, arsenic, vanadium, and aluminum, are associated with better survival [6]. *KRAS* mutation and its splice variants are involved in the control of calmodulin function in *KRAS*-driven PDAC [7]. To understand the mechanism and facilitate further study on PDAC, this review article focused on the various critical alterations that occur in PDAC at the RNA, proteins, and metabolite level associated with genetic alterations of PDAC. We propose that profiling of RNA modifications will be helpful for a precise diagnosis in the early stages of PDAC [8] to allow therapeutic interventions and potentially increase patient survival rate [9].

## 2. Liquid Diagnosis of PDAC via DNA Mutations

Generally, liquid biopsy allows early tumor detection and stage diagnosis without the need for a solid tumor biopsy sample [10]. Nucleotides can be extracted from the surgically excised tumor. Further, the fluids in the pancreatic cysts can be subjected to liquid biopsy that has a relatively high specificity and sensitivity compared with blood sampling [10,11,12]. Currently available biomarkers of PDAC include carbohydrate antigen 19-9 (CA 19-9) [10] and carcinoembryonic antigen (CEA) [11,12]. Reportedly, 10% of patients have levels that fall below the detection level because of the absence of antigen secretion [10]. Further, CEA concentration can be used to differentiate between high-risk patients with mucinous cysts from and those having non-mucinous cysts [11,12]. Although several biopsy methods have been applied, the currently available options are inadequate in terms of sensitivity and specificity for clinical application. Thus, further developmental studies are required.

Patients with PDAC often present with late symptoms, at which point only 10–20% of patients are eligible for surgical resection [13]. Reportedly, in addition to the big 4 driver mutations of *KRAS*, *TP53*, *CDKN2A*, and *SMAD4*, the mutations in critical genes involved in the development and maintenance of pancreatic tissues are associated with pancreatic cancer with variable penetrance [13]. These genes include breast cancer type 2 susceptibility protein (*BRCA2*), breast cancer type 1 susceptibility protein (*BRCA1*), ataxia telangiectasia mutated, serine/threonine kinase 11 (*STK11*), serine protease 1 (*PRSS1)*, which encodes a trypsinogen, MutL protein homolog 1 (*MLH1*), and partner and localizer of BRCA2 (*PALB2*) [13]. Although a recent approach using genomic and proteomic technologies has provided chances to understand the mechanisms of the pathogenesis and biology of PDAC, clinically reliable diagnostic biomarkers are not yet available to diagnose early disease [13].

**Figure 1:**
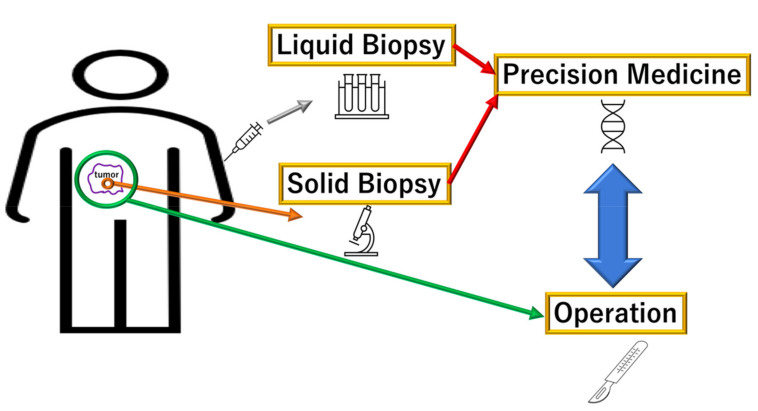
Biopsy procedure for precision therapy against pancreatic ductal adenocarcinoma (PDAC). The characteristics of tumors can be obtained by liquid and solid biopsy. Blood sampling and needle biopsy are performed to obtain liquid samples, whereas surgery, endoscopic approach, and needle biopsy, in some cases, are conducted to examine solid samples. The information obtained from liquid and solid biopsies will contribute to precision medicine. A multifaceted approach may improve patient prognosis in intractable cancers (e.g., PDAC).

The changes in 5-hydroxymethylcytosine (5hmC) in the circulating cell-free DNA (cfDNA) can be used to identify patients with PDAC via noninvasive detection methods [14]. Differential hydroxymethylation is observed most commonly in genes associated with pancreas development or function, including GATA-binding protein 4 (*GATA4*), GATA-binding protein 6 (*GATA6*), prospero homeobox protein 1 (*PROX1*), one cut homeobox 1 (*ONECUT1*), and Meis homeobox 2 (*MEIS2*), and in genes related to cancer pathogenesis, such as Yes1 associated transcriptional regulator (*YAP1*), TEA domain family member 1 (SV40 transcriptional enhancer factor) (*TEAD1*), *PROX1*, and insulin-like growth factor 1 (*IGF1*) [14]. The alterations in these genes are differentially enriched in PDAC along with the activation of *KRAS* and inactivation of *TP53*, suggesting that 5hmC changes can be used for the classification of PDAC even during the early stage [14]. Thus, further studies are warranted to improve the specificity and sensitivity of the liquid biopsy approach to detect PDAC in its early stages. At present, attempts are in progress to apply this process for the early diagnosis and metastasis diagnosis of PDAC as well as to select and monitor a treatment strategy by making complete use of multiple methods, such as surgery, chemotherapy, radiation therapy, and immune therapy, as combination [13] (Figure 1).

## 3. Diagnosis through Methods other Than DNA Sequencing

### 3.1. Metagenomics

**Figure 2:**
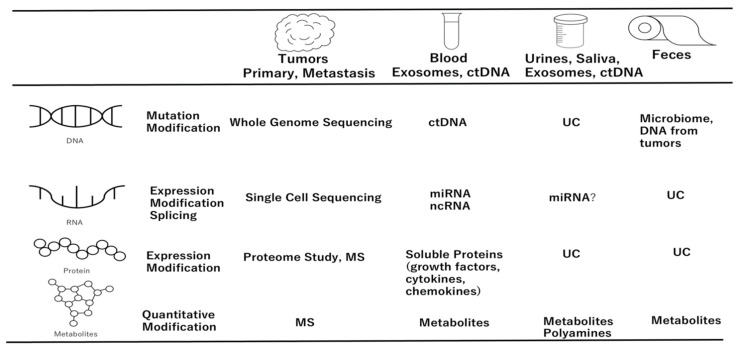
Multimodality approaches to diagnose and monitor pancreatic ductal adenocarcinoma (PDAC). Although various methods have been developed, no biomarker or PDAC diagnosis using only one method has been identified. The current situation is to increase the sensitivity and specificity by combining multiple methods. ctDNA, Circulating tumor DNA; UC, uncharacterized.

Oncogenic mutations in tumor tissues are suitable targets for the noninvasive screening. Numerous efforts have been made to detect tumor-derived cells, cell-free nucleic acids, and extracellular vesicles present in several body fluids, including urine and feces [15]. Several efforts have been made to develop efficient technology to detect PDAC [15,16,17]. A recent metagenomics study targeting the gut and oral microbiomes showed that it could provide a powerful source of biomarkers for identifying individuals with PDAC and their prognosis [16]. Significant enrichments of *Streptococcus* and *Veillonella* spp and depletion of *Faecalibacterium prausnitzii* were common gut signatures for PDAC in all three cohorts examined in the study [16]. The study identified 58 bacteriophages that could infect microbial species enriched in patients with PDAC in Japan, Spain, and Germany [16]. A genetically engineered PDAC murine model (KRASG12D/TP53R172H/PdxCre) and patient samples demonstrated that polyamine and nucleotide biosynthetic pathways were significantly elevated, indicating that the estimation of polyamine from the serum of patients with PDAC shows that total polyamine concentration in increased in the patients with PDAC compared to healthy volunteers, which is consistent with the estimation in PDAC murine model (KRASG12D/TP53R172H/PdxCre), and that serum polyamine levels in murine models (KRASG12D/TP53R172H/PdxCre) show an increased concentration of polyamines as tumors progress from early stages to PDAC [17]. The study suggests the potential usefulness of assessing microbial dysbiosis and polyamine metabolism as predictive biomarkers of PDAC in the early stages [17] (Figure 2).

### 3.2. Polyamines

A polyamine is an organic compound that has more than two amino groups. Polyamine metabolism is associated with the production of S-adenosylmethionine (SAM), a methyl donor for the methylation of critical substances, such as DNA, RNA, and histones [18]. Polyamine biosynthesis is frequently upregulated in cancer [18]. The enhanced flux of polyamines and their metabolites increases the levels of intracellular polyamines that are necessary for promoting cell growth and proliferation through intracellular biochemical mechanisms, such as the one-carbon metabolism in mitochondria and cell-to-cell interaction [18]. A previous study on polyamine metabolism analyzed human tissues and PAN 02 murine pancreatic cancer cells orthotopically injected into the pancreas of immune-competent C57Bl/6 mouse; the results revealed that polyamines play a major role in the development of PDAC [19] and in controlling the tumor microenvironment (TME) [20]. Higher expression of propolyamine genes was associated with poor patient prognosis, suggesting the usefulness of a polyamine blockade therapy against PDAC [20]. This observation is supported by the animal experiments on polyamine blockades, which resulted in decreased PDAC tumor weights and increased survival of mice [20]. Mechanistically, polyamine blockades led to macrophage infiltration and co-stimulatory marker (CD86) presentation on T cells, supporting the hypothesis that targeting polyamine metabolism can disrupt tumor progression by modulating TME and extending overall survival [20].

A urinary polyamine panel can distinguish between patients with human pancreatic cancer and pancreatitis and healthy controls [21], suggesting that a companion diagnostic approach may help treat PDAC. This study on cellular polyamine-dependent epigenetic regulations in human cancer cells demonstrated that polyamine flux (putrescine, spermidine, and spermine) suppresses histone lysine demethylases by competitive suppression against histone H3 lysine K4 (H3K4), active chromatin marking for many critical genes maintaining tissue homeostasis was shown to contribute to the development of several cancers [22]. In addition, previous studies have indicated that bone morphogenetic protein 9 (BMP9), an inhibitor of DNA binding 1, helix–loop–helix protein (ID1) signaling, promotes epithelial cell adhesion molecule (EpCAM)-positive cancer stem cell properties in gastrointestinal cancer, such as hepatocellular carcinoma; this supports the notion that polyamine flux enhances RNA expression of *ID1* in cancer stem cells [22,23]. Given that the critical enzyme in polyamine flux ornithine decarboxylase (ODC1) is involved in the maintenance of cancer stem cells and can be used for visualizing malignant cells with chemotherapeutic resistance [24,25], this enzyme may be a good candidate for use in the diagnosis, monitoring, and therapeutic approaches against malignant cancer. However, recent studies have indicated that polyamine metabolites are involved in oral squamous cancer [26], head and neck cancer [27], as well as colorectal cancer [28], which can be diagnosed or monitored using body fluids such as saliva. The big questions that remains to be solved is whether polyamine metabolites are secreted into the fluids directly or indirectly, i.e., through tumor–host interaction or other mechanisms.

### 3.3. Driver Mutations-Dependent Effects in PDAC

Among the big 4 driver mutations, *KRAS* plays a dominant role in the occurrence and development of PDAC in animals and humans (https://cancer.sanger.ac.uk/census (accessed on 1 August 2022)) [29,30,31]. Alterations in cyclin-dependent kinase inhibitors result in uncontrolled cancer cell growth by constitutive activation of cyclin-dependent kinases accelerating cell cycle progression from G1/S to G2/M through their checkpoints, which will give rise to strong proliferative phenotypes [32]. Alterations in the *TP53* gene in cancer cells, numerous functions of genome “guardians,” i.e., the maintenance of genome integrity, are applied. This function as “guardians” is involved in cell cycle control (arresting cell cycle and repairing damages) by transactivation of cyclin-dependent kinase inhibitor 1A (WAF1/SDI1/CIP1/P21) cyclin-dependent kinase inhibitor, as shown in the study on human PDAC tissues [32]. Recent studies on human PDAC cells reveal the roles of TP53 in multiple activation mechanisms of epithelial-to-mesenchymal transition (EMT), a critical phenotype for PDAC metastasis [33]. Furthermore, TP53 is demonstrated to be involved in the innate and adaptive immune regulation in TME of PDAC by concerting with *KRAS* mutation, as shown by the study involving leveraging in silico, in vitro, and in vivo models of human and murine PDAC [34].

Lastly, *SMAD4* is inactivated in 50% of PDACs, and its loss has been associated with worse overall survival and metastasis [35]. Mechanistically, *SMAD4* plays a role in signal transduction of the transforming growth factor-beta (TGF-beta) pathway, which is involved in inducing EMT [35]. SMAD4 loss in patients with PDAC was involved in the induction of EMT and further intermediary states, referred to as epithelial-mesenchymal plasticity, as shown by studies in human PDAC cells [35]. Taken together, the big 4 driver mutations are involved in the malignant phenotype of PDAC cells, such as altered cell growth, cell survival, cell cycle, or invasiveness, although the other relevant mechanism such as KRAS-dependent metabolism in tumors has emerged and are discussed in the next session.

#### 3.3.1. KRAS Proto-Oncogene GTPase (KRAS)-Dependent Phosphatidylinositol-4,5-Bisphosphate 3-Kinase Catalytic Subunit Alpha (PI3K), a Mechanistic Target of Rapamycin Kinase Pathway

Previous reports on human pancreatic cancers, cancer cell lines, and mouse models of PDAC have shown that KRAS mutations contribute to the activation of phosphatidylinositol-4,5-bisphosphate 3-kinase catalytic subunit alpha (PI3K)/v-Akt murine thymoma viral oncogene homolog 1 (AKT)/signaling pathway as well as serving as a mechanistic target of rapamycin kinase (mTOR) signaling pathway [36]. The silencing of the KRAS gene pathway is expected to inhibit the PI3K-Akt-mTOR signaling pathway to regulate EMT, proliferation, and apoptosis in cancer cells, as shown in a study on cancer tissues [37]. PI3K activated by the insulin receptor is mainly involved in glucose uptake into cells and the synthesis of proteins and glycogen [38]. PI3K catalyzes the reaction to produce phosphorylated phosphatidylinositol at the 3 position by phosphorylating inositol phospholipids [38]. PI3K activation results in various biological activities, such as cell differentiation, cell proliferation, metabolism, cell migration, and cytoskeleton reconstruction via molecules downstream of PI3K, as shown by in vitro analysis and in vivo models of human cancer cells [38,39]. This leads to the activation of the mTOR signaling pathway [39]. In this case, mTOR reflects the nutritional status of cells and regulates protein synthesis, cell proliferation, angiogenesis, and immunity, as indicated by studies involving in vitro cultures, three-dimensional models, and murine models of human cancer cells [40,41]. The mTOR inhibitor has been used as a stent restenosis prevention agent, an anticancer agent, and an immunosuppressive agent [40,41].

**Figure 3:**
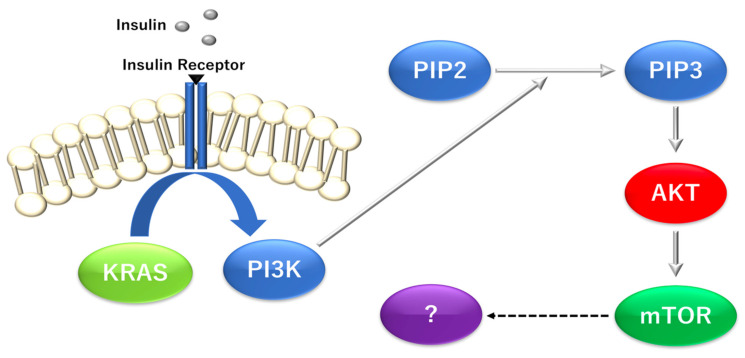
The mechanism by which KRAS proto-oncogene and GTPase (*KRAS*) mutations affect downstream signaling in the pancreatic ductal adenocarcinoma (PDAC). *KRAS*, KRAS Proto-Oncogene and GTPase; PI3Kl, phosphatidylinositol-4,5-bisphosphate 3-kinase catalytic subunit alpha; PIP2, phosphatidylinositol-4, 5-bisphosphate; PIP3, phosphatidylinositol-3, 4, 5-triphosphate; AKT, v-Akt murine thymoma viral oncogene homolog 1; mTOR, mechanistic target of rapamycin kinase. Under the control of KRAS, PI3 kinases catalyze the production of PIP3 by PIP2, although the downstream of mTOR has been studied for complete understanding.

Reportedly, the mTOR pathway is involved in the transcription of many genes in the sterol/cholesterol biosynthesis pathway, which are shared targets of the following transcription factors: sterol regulatory element binding transcription factor 1 (SREBP), SP1 transcription factor (SP1), and nuclear transcription factor Y subunit (NF-Y) in neurons [42] as well as programmed cell death 1 ligand 1/CD274 molecule (PD-L1) in gastric cancer organoids, which acts in a Hedgehog transcriptional effector glioma-associated oncogene homolog 1 (zinc finger protein) (GLI)-dependent manner [43]. mTOR is necessary to maintain mitochondrial oxidative function, and transcriptional complexes containing peroxisome proliferator-activated receptor gamma coactivator 1-α (PGC-1α) control mitochondrial oxidative function to maintain energy homeostasis in response to nutrient and hormonal signals [44]. The transcription factor yin-yang 1 (YY1) was identified as a common target of mTOR and PGC-1α, suggesting an important mechanism by which nutrient sensing affects the mTOR pathway and metabolic regulation through the transcriptional control of mitochondrial oxidative function [44]. Accordingly, clarifying the mechanism by which the mTOR pathway transcriptionally activates genes. In particular, the genes activated nutrient-deficient and low-oxygen environments, such as the cancer microenvironment observed in vivo, are expected to contain important target molecules regulated in the cancer microenvironment. Thus, the efficient suppression of such target genes is important for epoch-making drug discovery to control the KRAS-dependent and independent downstream signaling pathway, including PI3K and its related molecules (Figure 3).

#### 3.3.2. KRAS Proto-Oncogene and GTPase (KRAS)-Dependent Oncogene V-myc Avian Myelocy-Tomatosis Viral Oncogene Homolog (C-MYC) Pathway

The oncogene V-myc avian myelocytomatosis viral oncogene homolog (c-MYC) is the downstream target of KRAS. c-MYC interacts with several oncogenic and proliferative pathways in PDAC [45], and its overexpression is a common characteristic of PDAC. It predicts the aggressive behavior of cancer cells, as shown by in vitro cell culture and in vivo murine models of human cancer cells [45]. The KRAS and c-Myc pathways induce glycolysis dysregulation as represented by the Warburg effect, a biochemical phenomenon in cancer cells [46] and a hallmark of cancer [3,4]. The Warburg effect reveals the different metabolic but unique metabolic patterns with the characteristic of plasticity [47]. Interacting with retinoblastoma (RB)-associated protein 1 (E2F1), c-Myc induces genes involved in nucleotide metabolism and DNA replication [48,49]. In addition, microRNAs homeostatically attenuate the E2F1 expression [48]. The RB inactivation during cancer progression facilitates the G1–S transition of the cell cycle but also enhances malignancy characteristics, including drug sensitivity alterations [48,49]. In contrast, the RB inactivation enhances pro-inflammatory signaling through stimulation of the interleukin-6/STAT3 pathway, suggesting that the oncogenic KRAS and c-Myc pathways and tumor suppressive TP53 and RB pathways are collectively involved in the control of cancer metabolism [49]. Further, small molecules that hinder c-Myc-MAX heterodimerization or c-Myc/Myc associated factor X (MAX)/DNA complex formation can functionally inhibit c-Myc, implying that the targeting c-Myc can be achieved through transcriptional, post-transcriptional, and translational modifications in the treatment of PDAC [45].

**Figure 4:**
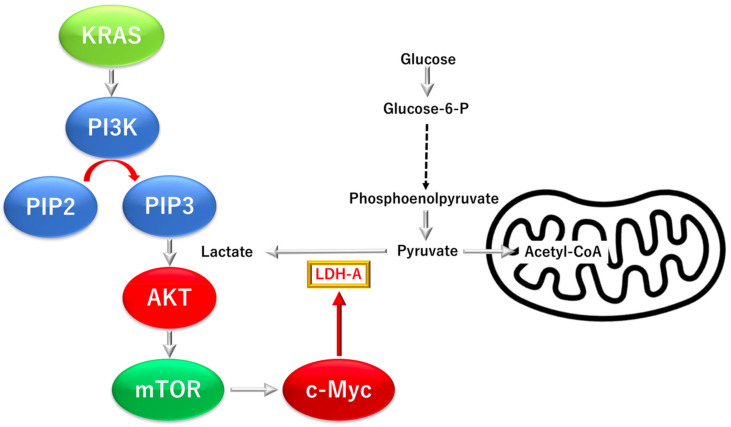
KRAS proto-oncogene and GTPase (KRAS) and oncogene V-myc avian myelocytomatosis viral oncogene homolog (c-MYC)-dependent glycolysis in pancreatic ductal adenocarcinoma (PDAC). c-MYC, oncogene V-myc avian myelocytomatosis viral oncogene homolog; glucose-6-P, glucose-6-phosphate; LDH-A, lactate dehydrogenase A subunit.

Under hypoxic conditions in TME, the hypoxia-inducible transcription factor HIF-1 cooperates with c-Myc to induce a transcriptional program for hypoxic adaptation [48]. Reportedly, c-Myc directly regulates the gene expression of glycolytic genes, including lactate dehydrogenase A [48]. Indirectly, c-Myc stimulates the expression of glutaminase protein and glutamine metabolism through the repression of miR-23a/b [48]. Collectively, the studies on in vitro biochemical assessments, cell culture, and in vivo murine models of human cancer cells demonstrated that the ectopic expression of c-MYC in cancers induces aerobic glycolysis and oxidative phosphorylation to provide sufficient energy and substrates to fulfill cell growth and proliferation in the context of TME [48] (Figure 4).

#### 3.3.3. KRAS Proto-Oncogene and GTPase (KRAS)-Dependent Alternative Tricarboxylic Acid Pathway

**Figure 5:**
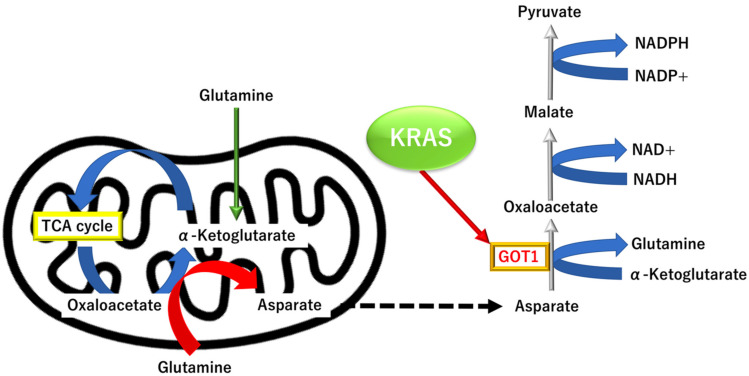
KRAS proto-oncogene and GTPase (KRAS)-dependent alterations of TCA metabolism in pancreatic ductal adenocarcinoma (PDAC). TCA, tricarboxylic acid.

Recent studies have indicated that the TME condition could elicit alterations in the metabolic flow of the tricarboxylic acid (TCA) cycle in the mitochondria of cancer cells [50,51,52,53,54,55,56]. First, the hypoxic conditions promote isocitrate dehydrogenase (IDH)-dependent carboxylation of α-ketoglutarate (α-KG) to citrate, leading to tumor cell growth and viability [50]. This suggests the role of this “oxidative pathway” for glutamine carboxylation by maintaining citrate synthesis and cell growth under hypoxic conditions. Second, under hypoxia in TME, a study on malignant cells indicated that the “reductive pathway” of the TCA cycle (i.e., glutamine metabolism by IDH1) mediates lipogenesis and production of acetyl coenzyme A, the central biosynthetic precursor to support fatty-acid synthesis and protein acetylation [51]. In addition, a study on human colorectal cancer indicated that the metabolism of isocitrate dehydrogenase-dependent carboxylation of α-KG to citrate is altered and produces oncometabolite D-2-hydroxyglurate (HG), which directly induced EMT in human cancer cells [52]. This finding is further supported by the mathematical analysis of imbalanced IDH1/2 expression associated with the 2-HG-inactivating β-oxygenation pathway in colorectal cancer [53]. Third, a study on PDAC cells indicates that KRAS-dependent metabolic pathway, in which glutamine supports PDAC cell growth, as shown by an in vitro and in vivo study [54]. In this PDAC-specific pathway, where the aspartate can be converted into oxaloacetate by aspartate transaminase (GOT1), oxaloacetate is converted further into malate and then pyruvate, which can increase the NADPH/NADP+ ratio to maintain the cellular redox state [54]. This supports the essentiality of this KRAS-dependent metabolic pathway in PDAC biology [54]. This pathway is dispensable in normal cells; therefore, it may be used to identify novel therapeutic approaches. Interestingly, the indispensable role of the KRAS-dependent metabolic pathway in PDAC is distinct from other tumors, such as colorectal cancer. Another study on *KRAS* mutations indicates their dispensable roles in colorectal cancer cells and suggests further the mechanism of metabolic adaptation to nutritional stress [55]. In colorectal cancer, a V600E mutation in v-raf murine sarcoma viral oncogene homolog B (*BRAF*) oncogene may attenuate the role of the KRAS-dependent metabolic pathway through AMP-activated protein kinase-mediated autophagy, leading to therapeutic resistance in cancer cells [56]. Thus, further studies are necessary to elucidate the role of KRAS in developing an innovative therapeutic approach against PDAC (Figure 5).

#### 3.3.4. KRAS-Dependent One-Carbon (1C) Pathway

**Figure 6:**
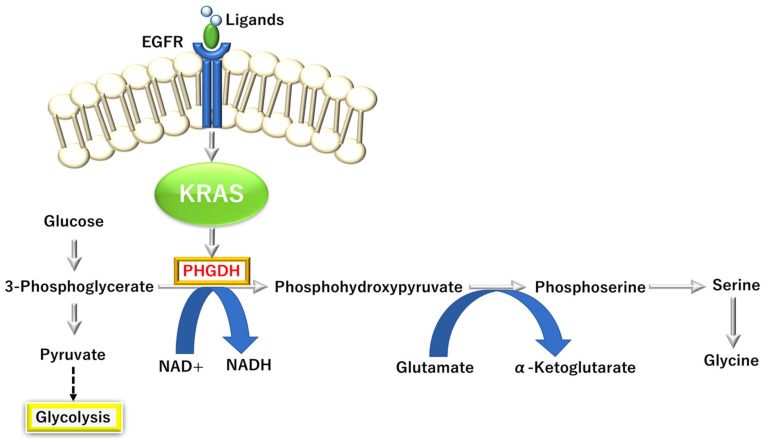
KRAS proto-oncogene and GTPase (KRAS)-dependent fueling serine and glycine metabolism in pancreatic ductal adenocarcinoma (PDAC). PHGDH, D-3-phosphoglycerate dehydrogenase; EGFR, epidermal growth factor receptor.

Recent studies indicate that in intrahepatic cholangiocarcinoma (iCCA), which can be influenced by surrounding cells in the liver. Epidermal growth factor receptor (EGFR) signaling and *KRAS* mutation (G12D) can activate interleukin 6 (IL6) production in iCCA cells [57]. This condition induces upregulation of phosphoglycerate dehydrogenase (PHGDH), the rate-limiting enzyme in the serine-glycine pathway in human iCCA, which correlates with euchromatic histone lysine methyltransferase 2 (*EHMT2*/*G9A*) expression [57]. In a G9A activity-dependent manner, KRAS mutation (G12D) promotes the PHGDH expression and glucose flow toward serine synthesis. It increases CCA cell viability [57], suggesting that the *KRAS* mutation can elicit metabolic reprogramming under inflammatory conditions in TME. Reportedly, the critical role of this KRAS-dependent serine biosynthesis pathway was demonstrated in PDAC, suggesting that targeting the serine biosynthesis pathway by inhibiting PHGDH is a potential therapeutic approach to eliminate PDAC cells in nutrient-deprived TME [58] (Figure 6).

Since serine and glycine are precursors of one-carbon (1C) folate metabolism, KRAS-dependent PHGDH expression is critical for the 1C folate metabolism-dependent synthesis of serine from glucose [59]. In highly proliferating cancer cells, dietary serine and serine de novo synthesis from glucose represents an important source of biomolecules for 1C folate metabolism [59]. In these cells, the 1C folate metabolism is activated and plays a role in the production of purines, a precursor of nucleotides, including DNA and RNA [59]. A previous study involving clinical samples determined that the status of enzymes of the 1C folate metabolism can predict the survival rate of patients with gastrointestinal cancer and provide the rationale for anticancer targets [60], such as methylenetetrahydrofolate dehydrogenase (MTHFD) 2, as shown by a study on human gastrointestinal cancer cells [61]. These studies further suggest the drugability of 1C folate as cancer diagnostic and therapeutic approaches and indicate that one of the promising targets is the MTHFD-2 [61,62].

**Figure 7:**
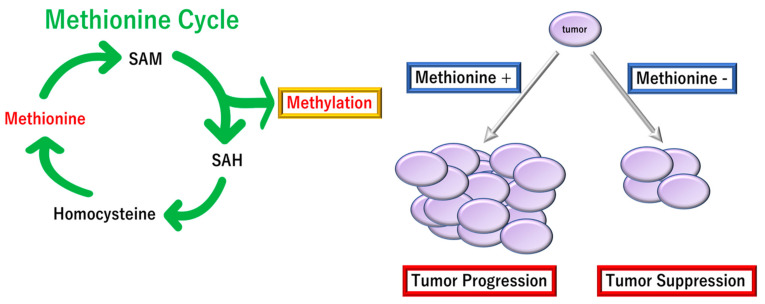
Competition of immune cells for nutrition in pancreatic ductal adenocarcinoma (PDAC). It has been suggested that some cancer cells are dependent on methionine (Hoffman effect). In the tumor microenvironment (TME), cancer cells are supposed to compete for methionine. SAM, S-adenosylmethionine; SAH, S-adenosylhomocysteine.

As resultant metabolites of 1C metabolism, S-adenosylmethionine (SAM) plays an indispensable role in the methylation of various targets, including DNA, RNA, proteins, and metabolites [63]. In TME, the methylated substances are involved in cellular metabolism; however, some of those can be secreted as intercellular communications to affect surrounding cells [64]. Previous reports indicate that >80% of cancer cells depend on methionine uptake in TME as the cancer cells can rapidly synthesize methionine from homocysteine, which will be consistent with cancer cells’ high demand or addiction to methionine for altered metabolic flux through pathway linked to SAM usages [65,66]. The mechanism also includes several processes involved by Cdc6 and prereplication complexes (breast cancer) [67], the nucleoside metabolism and polyamine synthesis (breast cancer) [68], and cycle arrest in G1 that functions through p38 mitogen-activated protein kinase (murine pre-B cells, human B lymphoblast cells, and human lung cancer cells) [69]. The Hoffman effect notes the methionine dependence of cancer, i.e., the stress sensitivity to deprivation and sufficiency of methionine in cancer [65]. This reinforces the mechanism of “zero-sum” competition in relatively closed areas as the poor vascularity of TME. Notably, in vivo evidence of tumor-dependency on dietary methionine was reported by Sugimura et al., who indicated that tumor growth in rats was significantly affected by the restriction of individual amino acids, such as methionine [70]. However, the magnitude of SAM synthesis’s dependence on the 1C folate metabolism or betaine in cancer cells remains unknown (Figure 7).

#### 3.3.5. Arginase 1-Dependent Immune Suppression in PDAC

A recent study indicated that neutrophil-like myeloid-derived suppressor cells (MDSCs) with high expression of CD13 can have immune suppression effects through arginase 1 expression in PDAC [71]. Given the heterogeneous characteristic features of MDSCs as potent suppressors of antitumor immunity, this study dissected MDSCs into subpopulations. MDSCs with high expression of CD13 suppressed more effectively T cell responses via an arginase-1-related mechanism [71]. Patients with PDAC with neutrophil-like MDSCs with high expression of CD13 had shorter overall survival, whereas monocyte-like MDSCs was less effective [71]. Although the signaling pathway from the tumor to MDSCs remains unclear, arginase 1 in MDSCs with high CD13 expression may consume arginine from the relatively closed TME, thus suppressing antitumor effector T cells [71]. This finding is in accordance with the fact that arginine plays a role in the modulation of T cell metabolism and enhances survival and antitumor activity [72,73]. Previously, CD13 (aminopeptidase N, which plays a role in reducing reactive oxygen species [ROS] after exposing chemotherapeutic reagents) was identified as a marker of cancer stem cells for gastrointestinal cancer [74]. Taken together, CD13 in MDSCs in TME may reduce ROS after exposing chemotherapeutic reagents and radiation and contribute to the survival of cancer cells, leading to a worse prognosis for patients.

### 3.4. Cancer-Associated Fibroblasts in PDAC

Cancer-associated fibroblasts (CAFs) do not contain mutations. However, tumor progression may entail a non-mutational conversion from tumor suppressive to tumor supportive, such as “education” of stromal TP53 [75]. Previous studies have shown that the neoplastic epithelium of PDAC exists within a dense desmoplastic stroma, a recognized critical mediator of disease progression through direct effects on cancer cells and indirect effects on the TME, including antitumor immunity, as shown by the studies on in vitro cell culture, in vivo murine models, and human tissues of PDAC [76]. Recently, reports on single-cell RNA sequencing in several cohorts of human PDAC tissues have been published [31,77,78,79,80,81]. By characterizing 136,000 single cells from more than 70 cases with PDAC, the study classifies myoblastic CAF (myCAFs) with a vital pathway of myofibroblasts and EMT and inflammatory CAF (iCAFs) with an inflammatory pathway [31,77]. Interestingly, the information of single-cell RNA sequencing emerged as a common strategy. Combined with analytical information on all expressed genes in the sample where they were expressed by spatial resolution to transcriptomics, and disease pathology research, a unique analysis of cell-cell interactions between cancer cells and host immune cells and fibroblasts in the cancer microenvironment has been clarified with spatiotemporal analysis, including position information and pseudo-temporal analysis. These profiling approaches will give rise to an efficient selection of therapeutic strategies upon individual patient information, which will contribute to optimizing therapy in patients with PDAC.

The current situation in clinical oncology is that PDAC remains among the diseases with the most urgent and prevalent medical needs. However, several therapeutic options have been performed, entering clinical trials as combination treatments [82]. It is expected that translational research focused on understanding the complicated connections among single cells, such as CAF in the TME, is increasingly valuable to innovate novel treatment approaches [82]. Recent studies revealed the heterogeneity of CAF, including myCAFs and iCAFs, and antigen-presenting CAFs (apCAFs), which show major histocompatibility complex II (MHC II) family genes, as an attractive target for immune therapy [83]. Moreover, the critical roles of the metabolic state of CAFs emerged, given that a population of CAFs is dependent highly on glycolysis. In contrast, the cancer cells used oxidative phosphorylation as a significant metabolic mode rather than glycolysis, suggesting the critical role of intercellular communication between cancer cells and CAFs in controlling PDAC [84].

## 4. PDAC Detection and Monitoring by RNA Profiling

### 4.1. isomiR and episomiR

MicroRNAs (miRNAs) are short nonprotein-coding RNAs with an average length of 22 nucleotides (nt) and are involved in post-translational control by binding to the 3′-untranslated regions (UTR) of genes to degrade mRNAs or inhibit translation [85]. By expression profiling, miRNAs’ potential in cancer diagnosis has been previously demonstrated [85]. Since then, miRNAs have been known to be useful in the detection and monitoring of cancers, including PDAC [86]. The combination of miRNAs with the standard tumor markers has increased the sensitivity and specificity of biomarkers in liquid biopsy [87]. Furthermore, a study on miRNAs allowed the identification of a novel mechanism of TGF-beta/activin that induces EMT and stemness in PDAC [88], suggesting that miRNAs are functionally crucial for PDAC pathogenesis.

Recent studies on the development of new high-throughput technologies (e.g., deep RNA sequencing) have facilitated the discovery of miRNA isoforms, i.e., variations with respect to the reference sequence named isomiRs through studies on human, murine, and other species [89]. Studies on isomiRs indicated the tissue-specific distribution of the precursors and mature sequences, the genomic distribution of uncharacterized miRNA genes and identification of new clusters, and isomiR characterization, suggesting that many miRNAs were regulated in a tissue/organ-specific manner in animals [90].

The recent study on miRNAs involving mass spectrometry demonstrated that the methylation of adenine and cytosine occurs in patients with PDAC but not in healthy participants [91]. Thus, SAM-dependent RNA methylation is a good candidate for biomarkers in liquid biopsy [91]. Analysis through another method, i.e., current tunnel sequencing, revealed cancer-specific methylation of miRNAs [92], suggesting that the epitranscriptomic modification of miRNAs, named eisomiRs, can provide the potential for biomarkers [93]. In PDAC, the methylation of adenosine of polo-like kinase 1 (*PLK1*) plays a role in regulating cell cycle homeostasis as a potential target of radiotherapy in PDAC [94]. These studies suggest that RNA modification will be essential for the detection and monitoring of miRNAs as well as for the mechanistic studies on target genes [94].

### 4.2. circRNAs

Long noncoding RNA (lncRNA) is a nonprotein-coding RNA with a length longer than 200 nucleotides (as reviewed in reference [95]). Among different classes of lncRNAs, circular RNAs (circRNAs) are formed by back splicing events. They are stable in circulation due to the unavailability of free ends for RNase to degrade circRNAs [96]. A recent study showed that circNFIB1 (*hsa_circ_0086375*) inhibits lymphangiogenesis and lymphatic metastasis by binding to *miR-486-5p*, which regulates phosphoinositide-3-kinase regulatory subunit 1 (PIK3R1) and vascular endothelial growth factor C (VEGF-C) axis in PDAC. As shown by in vitro and in vivo experiments on human PDAC cell lines (PANC1, Capan-2, and SW1990) and human pancreatic ductal endothelial cells (HPDE), which revealed that circNFIB1 is negatively associated with lymph node metastasis in PDAC [97]. Another example is the circ-RNAs *CircEYA3*, which induces energy production to promote PDAC progression through the *miR-1294*/c-myc axis by studying human PDAC cells (Capan-1, MiaPaCa-2, SW1990, PANC-1, BXPC-3, and CFPAC-1) and HPDE cells [98]. These two studies exemplify that circRNAs often connect with other noncoding RNAs or miRNA axes, suggesting that circRNAs exert a sponge function for the other macromolecules.

## 5. PDAC Detection and Monitoring by Volatile Organic Compounds

A recent study on volatile organic compounds (VOCs) by gas chromatography–ion mobility spectrometry and gas chromatography-time-of-flight mass spectrometry allowed the identification of several cancer-specific substances in the urine of patients with PDAC, including 2,6-dimethyl-octane, nonanal, 4-ethyl-1,2-dimethyl-benzene, and 2-pentanone [99]. Similarly, analysis of urine samples of patients with hepatocellular carcinoma revealed the presence of 4-methyl-2,4-bis(p-hydroxyphenyl)pent-1-ene (2TMS derivative), 2-butanone, 2-hexanone, benzene, 1-ethyl-2-methyl-, 3-butene-1,2-diol, 1-(2-furanyl)-, bicyclo(4.1.0)heptane, 3,7,7-trimethyl-, [1S-(1a,3β,6a)]-, and sulpiride [100]. In addition, a study urine from patients with prostate and bladder cancers identified toluene; methyl isobutyl ketone; dodecane; phenol; cyclopentanone, 2-methyl-; 2-hexanone; heptanal; p-xylene; nonane, 3-methyl-; tetradecane; nonanal; biphenyl; acetic acid; 2-pentanone [101].

Recently, studies beyond machine application (e.g., regular MS) were conducted using nematodes. A study used nematodes for detecting PDAC in the early stages using urine samples and showed that this method had higher sensitivity compared with existing diagnostic markers such as CA 19-9 and CEA [102]. This finding showed that biological reactions observed in the form of worms’ behaviors could help differentiate between samples from patients and healthy volunteers, suggesting that the worms can be used to detect uncharacterized substances, which can go undetected in standard mass spectrometry analysis.

## 6. Conclusions

At present, tumor removal by surgery is the best choice to improve the survival of patients with PDAC. Thus, it is necessary to find the signs as early as possible, diagnose the early stages of PDAC, and consider performing the surgical operation. Chemotherapy, radiation therapy, and immunotherapy will support therapeutic approaches to treat patients with PDAC. As we discussed here, to achieve the diagnosis in the early stages, recent studies emerged on the significance of multiple approaches, including metagenomics (intestinal bacteria), metabolomics (polyamines), and transcriptomics (RNAs). As the proof-of-concept, those mechanisms are involved directly and indirectly in the big 4 driver mutations. The recent approaches extend to understanding intercellular communications (among CAFs, immune cells, and cancer cells) in TME, suggesting that the spatiotemporal single-cell analysis will be able to realize highly individualized cellular level therapies. Those findings may lead to the innovation of novel treatments for advanced stages.

Moreover, as monitoring methods, recent state-of-art approaches suggest that the VOC and its related substances are involved in cancerous conditions of PDAC. The new methods of diagnosis could add new modalities that machines such as regular MS cannot reach. Further research on this topic is warranted. Although DNA mutations are known causes of various cancers, examining molecular signatures beyond DNA, as outlined in this review, may shed light on curing intractable cancer, such as PDAC.
